# Release of sugars and fatty acids from heavy oil biodegradation by common hydrolytic enzymes

**DOI:** 10.1038/s41598-019-51796-4

**Published:** 2019-10-30

**Authors:** Michael Mislan, Ian D. Gates

**Affiliations:** Department of Chemical and Petroleum Engineering Schulich School of Engineering University of Calgary, Calgary, Canada

**Keywords:** Hydrolases, Carbon cycle

## Abstract

In response to recent advances in understanding relating to the remarkable persistence of soil organic matter during burial and diagenesis, we examine the extent to which bitumen compositionally reflects the soil organic matter from which it was derived. Through a simple set of experiments, exposure of bitumen to lipase and cellulase, two enzymes effective in the biodegradation of soil organic matter, resulted in the release of glycerin, palmitic and oleic fatty acids from lipase digestion in addition to the release of glucose, alkylphenols and acyclic polyols from fermentation with cellulase, consistent with the products expected these enzymes. These results are significant in that they suggest that heavy oils are more similar to their soil precursor than previously thought, that biodegradation of bitumen can be accelerated using common over the counter enzymes in aerobic conditions and that heavy oils, which are 1000 times more abundant than coal, can release similar biomolecules as those generated in bioreactor culture or biomass harvest, using two of the most abundantly produced enzymes presently available.

## Introduction

Bitumen, kerogen and heavy oils are terminal products of the biogeochemical carbon cycle whereby naturally generated organic molecules deposited in aquatic sediments are decomposed into humic matter then progressively transformed into macromolecular fossils over geological timescales following burial^1–3^. Together they are the second-largest carbon sink on earth behind carbonate mineral deposits^4,5^ even though the majority of all deposited organic litter is remineralized or otherwise refluxed back into the biosphere instead of being eventually stored as sedimentary organic matter^6–8^. More recalcitrant biomolecules tend to accumulate in soil organic matter to form humic compounds in the process of pedogenesis including aliphatic lipids^9^, steroids^1^, chlorophyll^10^ and phenolic biomass^11^. Over millions of years this biomolecular mixture has progressed towards thermodynamic equilibrium under the forces of burial stress, entropy, thermal maturation, biodegradation, contact with water and minerals, and spontaneous recondensation to form supramolecular structures with both biotic and abiotic moieties^12–15^.

As a consequence of the fact that kerogen is the diagenic maturation product of soil organic matter, it is possible to classify heavy oils with respect to the ecological conditions which generated their source rocks with particular biochemical precursors. While asphaltenes and the kerogens from which they derive are largely considered to be the defunctionalized and polycondensed carbon skeletons of their original biochemical inputs, the first evidence that persistent biological moieties can be found in petroleum hydrocarbon deposits was identified in 1934 when the existence of Chlorophyll-derived Porphyrin groups was discovered in crude oil^10^. Since then many largely unaltered biomarkers have been discovered in kerogens and bitumen, either protected by occlusion inside or integration with a larger macromolecule^14,16^ or through association with a mineral phase^17,18^. Type I and II heavy oils contain a higher proportion of compounds derived from algal lacustrine or planktonic marine biomass respectively and are generally characterized by a higher lipid-content in the biotic source material^19^. These inputs have often left enough ester bonds in polar asphaltene moieties that many heavy oils can be susceptible to saponification^20–22^ and related compounds such as n-alkanoic acids, steroids, and cholesterol have been released with various chemical and thermal methods^16,23–25^. Type III heavy oils derive from a source material containing terrestrial plant matter, associated with the presence of polyaromatic macromolecules such as lignin, terpenes and cellulose^26^, which is the single most abundant biopolymer on earth^27,28^. These precursors have resulted in the detection of ether bonds in polar components of bitumen^20,29,30^.

However, prior to this study it has always been assumed that heavy oils differ from soil organic matter to such an extent that they are largely recalcitrant to biological means of degradation, having already undergone extensive biodegradation *in-situ* and that these biomarkers can only be separated from the hydrocarbons using chemical and/or thermal treatments. Only recently has research suggested that more labile biomolecules including fatty acids and cellulosic sugars can persist inside soil organic matter macromolecules in a relatively unaltered state^4,17,31–33^. Athabasca bitumen is thought to derive from a mixture of Type II and III kerogens^34^ and as a consequence may be amenable to digestion by both lipid-degrading lipase and cellulose-degrading cellulase. Therefore this is the first study to investigate whether hydrolytic enzymes able to cleave lipid-derived ester and cellulosic ether bonds in soil organic matter^35–37^ also affect bitumen. Lipase and cellulase are two of the most abundant industrially-produced enzymes on earth and the ability to enzymatically biodegrade bitumen to release readily bioavailable compounds has the potential to act as a foundational tool for future heavy oil remediation and bioengineering processes.

## Results and Discussion

**Figure 1:**
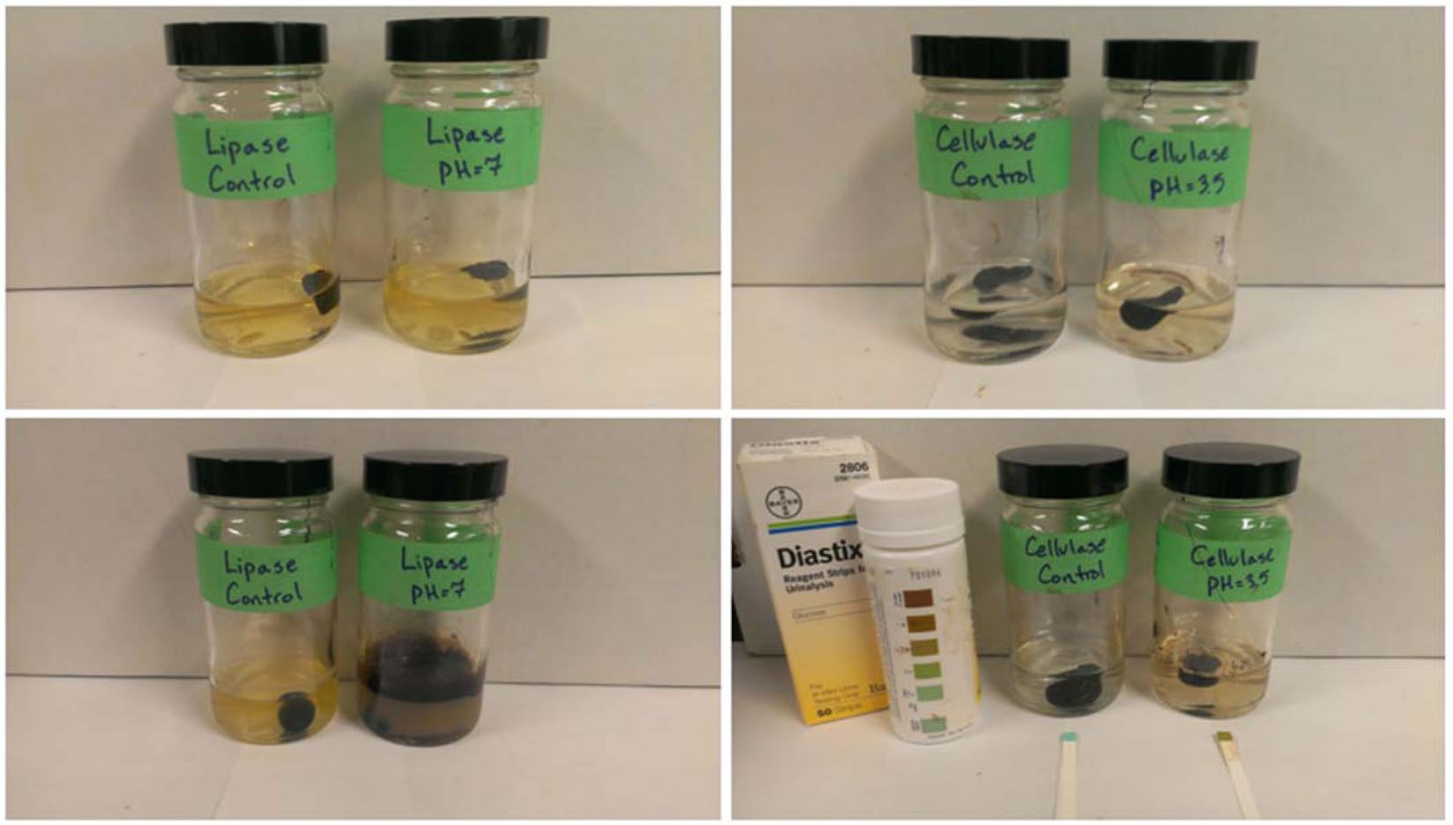
Lipase and cellulase mixing experiments conducted on Athabasca bitumen. Lower images demonstrate relative change in enzyme-fermented samples after 90 hours of agitation in aerobic conditions. The cellulase-fermented sample on the lower right hand side measures positive when tested with a commercial urinalysis glucose test strip.

As shown in Fig. 1, bitumen placed in aqueous enzyme solution and left in aerobic, agitated jars at room temperature broke down over a period of days to weeks into smaller fragments and produced turbidity changes relative to controls. Asymptotic change is observed after a week in agitated fermentation, suggesting the reaction may be limited by a combination of product inhibition, enzyme deactivation and depletion of available active sites^38–40^ but more study is required.

**Figure 2:**
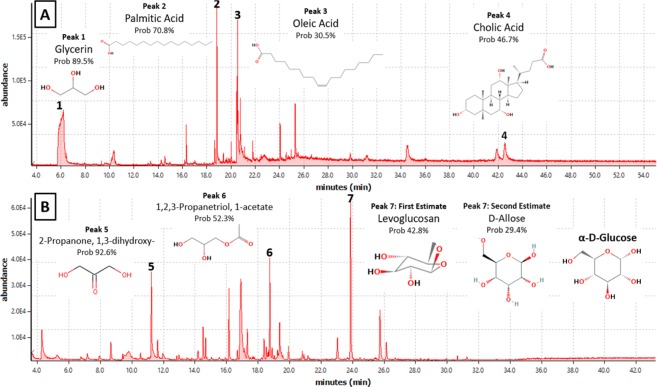
GC-MS results of aqueous enzymatic bitumen biodegradation products by (**A**) lipase and (**B**) cellulase.

GC-MS (Agilent 5975 GC-MSD) analysis of the aqueous phase suggests that many enzymatic bioproducts were created which are consistent with the expected products of these enzymes. Samples digested with lipase contained glycerin and two large peaks, displayed in Fig. 2, which were identified by GC-MS as C16 palmitic and C18 oleic fatty acids, also known as palm and olive oil, respectively. This is consistent with the first study to ever identify intact n-alkanoic fatty acids in bitumen by Peng *et al*. which found that cleavage of ester bonds in bitumen by saponification released a range of even carbon-numbered n-alkanoic acids between C12-C24 with the two largest peaks corresponding to C16 and C18^41^, a finding corroborated by similar studies^20,42^. This result is also consistent with the fact that these are two of the most abundant fatty acids found in plants, animals, and microorganisms^43^. However, these results are unique in the literature with respect to the identification of Glycerin amongst the compounds released by ester bond hydrolysis. This suggests the fatty acids may have been present in the bitumen in the form of glycerides which are esters of glycerol bonded to fatty acids. Glycerides or acylglycerols are ubiquitous in naturally produced fats and the fact that these are hydrolyzed into glycerin and fatty acids by microbial lipases is entirely consistent with their known mechanisms of catalytic action^44^. Interestingly while most biomarker studies which produced n-alkanoic acids find broad distributions of fatty acids between a range of 10 or more carbon numbers^20,41,42,45^ the results of this study are unique in that only C16, C18 and glycerin were identified as significant peaks, also suggesting the lipase hydrolysis mechanism is exceptional in contrast to chemical or pyrolytic methods of ester cleavage. In addition to the fatty acids and glycerin, a smaller amount of diverse organic molecules appear to have been released by the lipase hydrolysis, mostly comprised of acyclic polyols with higher carbon numbers than glycerin and some ester-functionalized aromatic compounds. However, given the inclusion of ox bile acid salts in the digestion solution to assist the lipase hydrolysis, largely reflected by the presence of a cholic acid peak, and the lower match probabilities of these less abundant compounds when comparing their spectrograms to the GC-MS library, a more detailed examination of these other degradation products must be left to more detailed analytical studies. Many of the oxygen-containing moieties which present viable active sites for the enzymes studied reside in aromatic and acyclic polar fractions of bitumen which partition themselves between the maltene and asphaltene phases^20,33,41^. It has been suggested that these moieties acting as sidechains to asphaltene micelles may play a significant role in the stabilization of the colloidal structure of bitumen^20,46–48^ which might account for the observation that lipase-degraded bitumen samples break up under agitation relative to controls.

Cellulase digestion appears to have produced a range of alkylphenols and acyclic polyols, many of which contain hydroxyl, carboxyl, carbonyl, ester and ether functional groups. Results from GC-MS analysis, displayed in Fig. 2, indicated a peak associated with the presence of a molecule resembling Levoglucosan, a sugar-type molecule often produced alongside glucose from biomass pyrolysis^49^, or D-Allose, a rare glucose stereoisomer^50^. Nonetheless the cellulase-upgraded bitumen solutions test positive when contacted with commercial glucose strips (Beyer; ModelID2806) suggesting the presence of a compound which can react to become gluconic acid^51^. While prior studies investigating degradation products released by bitumen produced by ether bond cleavage have also found alkylphenols^20^ as well as hydroxyl and carbonyl groups^30,41,52^ this study is the first to report the presence of intact, sugar-type molecules in heavy oil. While these results are surprising and require further inquiry, it should be noted that they are consistent with the products that would be expected from the biodegradation of biomass or soil organic matter using cellulase^37^. This discovery may also support the results of Mesle *et al*. which found microbial phenotypes known to metabolize sugars and fatty acids as part of a microbial consortia biodegrading kerogen *in-situ*^53^.

**Figure 3:**
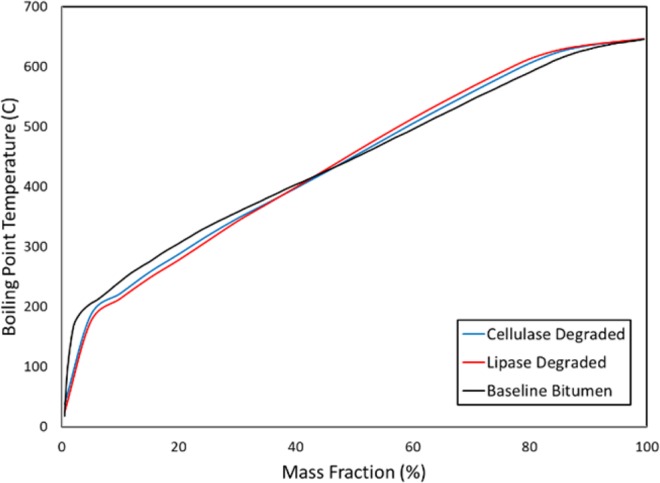
Simulated distillation results of original, unaltered bitumen compared to enzyme degraded samples demonstrating an alteration of their molecular weight distributions.

Simulated distillation was used to compare the molecular weight distribution of the bitumen before and after enzymatic biodegradation, shown in Fig. 3. For both enzymes tested, it was found that compounds towards the middle of the molecular weight distribution were degraded to generate smaller and larger molecules at the same time. The formation of larger bitumen molecules suggests the hydrolytic enzyme biodegradation may be a similar process to an accelerated maturation of sedimentary organic matter *in-situ* whereby some microbial biodegradation products undergo spontaneous condensation reactions to become sidechains of larger macromolecules^1,12^. Analysis of the simulated distillation boiling point curves suggests that up to 4 wt% of the bitumen molecular weight distribution is altered by lipase digestion while approximately 2 wt% was affected by cellulase degradation. These results are within a similar range as those found by other biomarker studies involving the cleavage of ester and/or ether bonds, which range from around 1–2wt%^20^ up to yields as high as 6 wt%^41^ to 11 wt%^52^ depending on the bitumen and method of bond cleavage. However, it should be noted that all of these values are highly approximate given the considerable experimental difficulty involved in analyzing heterogenous heavy oil mixtures.

The extent to which bitumen and other heavy oils can be biodegraded has been debated for over 50 years and many studies have been performed to test the biodegradative potential of hydrocarbons under various conditions. Slow kinetics and an effective biodegradative molecular size limit of C44 has been observed in anaerobic reservoir conditions containing indigenous microbial communities^13,54,55^ which have adapted to nutrient-deprived, growth arrested phenotypes over diagenesis^56^. Studies which have succeeded in biodegrading asphaltenes with microbial cultures have always done so under aerobic conditions^57–59^. Prior studies have offered hints that hydrolytic enzymes could potentially be used biodegrade petroleum compounds. Lipase activity has been used as an indicator of microbial activity during the biostimulated remediation of diesel in soils, but it was asserted that these lipases only reacted with products of otherwise unspecified hydrocarbon biodegradation reactions^60^. While the majority of biological enhanced oil recovery research has focused on attempts to modify wettability^61,62^, at least one successful field trial in Myanmar was conducted using a hydrolytic enzyme to biodegrade paraffins *in-situ*^63^. Another researcher in China has reported the biodegradation of crude oil by a fungal enzyme mixture prepared from a pseudomonas species which reportedly contained lipase^64^. There are even a number of research groups currently biodegrading asphaltenes with a range of other enzymes which have been isolated and enriched from indigenous heavy oil degrading communities^59^ including some which were chosen specifically for their lignocellulytic ability^65^.

Soil organic matter is the source material for asphaltenes and the results of this study emphasizes the fact that oil shares a continuum with soil. Cellulase and lipase were tested as they reflect almost universal inputs to soil organic matter and the results presented in this study suggest that what appear to be recalcitrant hydrocarbons can potentially be biodegraded under the right conditions depending on the extent to which they reflect their biological source. Humic and proto-kerogen materials have been generated from benchtop *ex-situ* decay and maturation of living organic matter such as plant tissue^66^, scorpion carcasses^67^, and algae^68^. However, it is an open question regarding at exactly what point these geochemical fossils transform into wholly abiotic carbon structures that are entirely recalcitrant to microbial activity more similar to graphite. It is possible that results demonstrating the persistence of relatively bioavailable moieties in soil organic matter^3,4,6,7,12,32^ might extend to kerogens and bitumens to a greater extent than might be expected considering millions of years of oxidation, entropy and weathering.

One of the key constraints preventing the global chemical industry transitioning away from its petrochemical foundation to a biochemical one regards how to scale up the procurement of biological feedstock streams with minimal ecological impact. Therefore, this technique could present an opportunity to not only remediate heavy oil spills but derive value from abandoned petroleum reservoirs by providing an alternative source for bioproducts such as palm oil while avoiding further deforestation. This study shows that it may be possible to partially convert heavy oils and kerogen *in-situ* into similar biomolecules as those generated in bioreactor culture or biomass harvest, using only oxygen and two of the most abundantly produced enzymes available on the market at this moment. All oils are derived from soil, but some are more like soil than others and this knowledge can guide a different approach to petroleum bioengineering which regards hydrocarbons as part of the continuum of nature rather than something outside of it. It stands to reason that bitumen, kerogen and all hydrocarbons are not only an ultimate thermodynamic sink for carbon-based life, they are also a plentiful biological carbon source which can potentially be transformed and refluxed back into the biosphere instead of the atmosphere.

## Methods and Materials

Bitumen was sourced from the McMurray Formation in the Athabasca oil sands deposit in Northern Alberta. The bitumen was produced by steam-assisted gravity drainage and passed through a central processing facility where it was separated from an emulsion with water by contact with surfactants but otherwise was untreated or altered prior to placement in the enzyme solution. Cellulase produced by *Aspergillus niger* (MP Biomedical; CAS No. 9001-32-1) and Lipase from *Candida rugosa* (MP Biomedical; CAS No. 9012-54-8) were purchased commercially for use in this study. Cellulase solutions were prepared by combining 20 mL deionized (DI) water with 0.35 mL Acetic Acid (VWR, CAS No. 64-19-7, 0.998–1.002 N), 0.1 g Sodium Bicarbonate (VWR, CAS No. 144-55-8, ACS Purity ≥99.7%) and 0.1 g Cellulase followed by the addition of bitumen. Lipase solutions were prepared by mixing 20 mL DI water with 0.1 g Sodium Bicarbonate, 0.1 g Purified Ox Bile Acid (HiMedia Laboratories, CAS No. 61001-534, ≥45% Cholic Acid content) and 0.1 g Lipase followed by the dropwise addition of Acetic Acid until a pH of 7 was measured before bitumen was added. All tests were conducted on a reciprocating shaker at atmospheric pressure (~92 kPa) and room temperature (22 °C).

GC-MS analysis was conducted on an Agilent 5975 GC-MSD. GC-MS samples were prepared by drying the aqueous solution under ambient condition then dissolving the precipitant in methanol. Simulated distillation was conducted using an Agilent 7890B gas chromatograph using the ASTM D2887 test method with a carbon disulfide solvent.

## Data Availability

All data reported in this article are available from the corresponding author.
